# Association between Congenital Cytomegalovirus Infection and Brain Injury in Neonates: A Meta-analysis of Cohort Studies

**DOI:** 10.1155/2021/9603660

**Published:** 2021-10-15

**Authors:** Li Zhang, Zhankui Li, Xiang Han, Hongyan Du, Yingli Cao, Yingmei Liu, Wenfeng Wang

**Affiliations:** ^1^Department of Neonatology, Northwest Women's and Children's Hospital, Xian 710061, China; ^2^Department of Obstetrics, Northwest Women's and Children's Hospital, Xian 710061, China; ^3^Department of Pathology, Northwest Women's and Children's Hospital, Xian 710061, China; ^4^Xian Medical University, Xian 710021, China; ^5^School of Science, Shanghai Institute of Technology, Shanghai 201418, China; ^6^International Academy of Visual Art and Engineering, London E16 1AH, UK; ^7^Interscience Institute of Management and Technology, Bhubaneswar 752054, India; ^8^RCEECA, Chinese Academy of Sciences, Urumqi 830011, China

## Abstract

**Objective:**

To assess association between congenital cytomegalovirus (CMV) infection and brain injury in neonates.

**Methods:**

The literatures from inception to November 4, 2020, were searched through PubMed, Embase, Cochrane Library, and Web of Science. Heterogeneity test was conducted for each indicator and measured by *I*^2^ statistics. If *I*^2^ ≥ 50%, the random effects model was applied; otherwise, the fixed effects model was used. Sensitivity analysis was performed for all models. Weighed mean difference (WMD) was used as the effect size for measurement data, and risk ratio (RR) was as the effect indicator.

**Results:**

A total of 13 studies, including 4,262 congenital CMV infection neonates, were enrolled in this study. Our results showed that the rate of hearing impairment (RR: 2.105, 95% CI: (1.115, 3.971), *P* = 0.002), sensorineural hearing loss (SNHL) (RR: 17.051, 95% CI: (6.201, 46.886), *P* < 0.001), and microcephaly (RR: 2.283, 95% CI: (1.325, 3.935), *P* =0.003) in neonates infected congenital CMV was higher than that in control group.

**Conclusion:**

The risks of hearing impairment, SNHL, and microcephaly in neonates during childhood may be associated with congenital CMV infection. It is necessary to establish neonatal screening programs and comprehensive diagnostic tests for patients to reduce the risk of adverse brain damage to the congenital CMV infection as early as possible and to improve the prognosis of the newborn.

## 1. Introduction

Congenital cytomegalovirus (CMV) infection refers to an infectious disease caused by vertical transmission of CMV in the fetus due to the mother's CMV infection during pregnancy, which causes damage to multiple organs of the fetus or newborn [1, 2]. After pregnant women are infected with CMV, the vertical transmission rate is as high as 32%-40%, with the prevalence of congenital CMV infection worldwide in live born newborns is 0.5%-3% [2]. CMV in pregnant congenital CMV infection women causes intrauterine infection of the fetus through the placenta, which is the most important cause of congenital central nervous system damage caused by intrauterine infection. CMV infected infants may have severe brain damage, causing brain dysfunction, such as severe decreases in cognitive capacity, mental retardation, and seizures [3–5]. Insights into the risk of congenital CMV-associated impairment can help optimize care of neonates infected with congenital CMV and stimulate preventive measures to improve the prognosis.

Several studies showed congenital CMV infection patients develop long-term sequelae, including sensorineural hearing loss (SNHL) and neurodevelopmental damage, ranging up to severe decreases in cognitive capacity, which is often irreversible [3, 4, 6–8]. Nevertheless, there is a study that shows that the risk of developing SNHL after age 5 years among case-patients was not different than in uninfected children [9]. A prospective follow-up study indicated that full-term infants with postnatal congenital CMV infection are often asymptomatic, without long-term sequelae or hearing problems [10].

There were studies attempting to investigate association between perinatal infections and neonatal brain injury [11, 12]. Nevertheless, studies estimating brain injury of congenital CMV infection in neonates were limited. Herein, we performed this meta-analysis to evaluate association between congenital CMV infection and brain injury in neonates so as to start a comprehensive screening of newborns with congenital CMV infection as soon as possible and achieve early intervention, comprehensive treatment, and dynamic monitoring to reduce the complications and sequelae caused by congenital CMV infection in newborns.

## 2. Methods

### 2.1. Search Strategy

The literatures from inception to November 4, 2020, were searched through PubMed, Embase, Cochrane Library, and Web of Science. The search words were as follows: “Virus Diseases” OR “Disease, Virus” OR “Diseases, Virus” OR “Virus Disease” OR “Virus Infections” OR “Infection, Virus” OR “Infections, Virus” OR “Virus Infection” OR “Viral Diseases” OR “Disease, Viral” OR “Diseases, Viral” OR “Viral Disease” OR “Viral Infections” OR “Infection, Viral” OR “Infections, Viral” OR “Viral Infection” OR “rubella virus” OR “cytomegalovirus” OR “hepatitis B virus” OR “herpesvirus” OR “human papilloma virus” OR “HPV” OR “Human parvovirus B19” OR “B19” OR “human immunodeficiency virus” OR “coxsackie virus” AND “Brain Injuries” OR “Injuries, Brain” OR “Brain Injury” OR “Injury, Brain” OR “Injuries, Acute Brain” OR “Acute Brain Injuries” OR “Acute Brain Injury” OR “Brain Injury, Acute” OR “Injury, Acute Brain” OR “Brain Injuries, Acute” OR “Brain Lacerations” OR “Brain Laceration” OR “Laceration, Brain” OR “Lacerations, Brain” OR “Brain Injuries, Focal” OR “Brain Injury, Focal” OR “Focal Brain Injury” OR “Injuries, Focal Brain” OR “Injury, Focal Brain” OR “Focal Brain Injuries” OR “Hearing Loss, Sensorineural” OR “Sensorineural Hearing Loss” OR “Hearing Loss, Cochlear” OR “Cochlear Hearing Loss” OR “SNHL” OR “brain” OR “Encephalon” OR “neurodevelopment” AND “Fetus” OR “Fetuses” OR “Fetal Structures” OR “Fetal Structure” OR “Structure, Fetal” OR “Structures, Fetal” OR “Mummified Fetus” OR “Fetus, Mummified” OR “Retained Fetus” OR “Fetus, Retained” OR “Fetal Tissue” OR “Fetal Tissues” OR “Tissue, Fetal” OR “Tissues, Feta” OR “Infant, Newborn” OR “Infants, Newborn” OR “Newborn Infant” OR “Newborn Infants” OR “Newborns” OR “Newborn” OR “Neonate” OR “Neonates.”

### 2.2. Inclusion and Exclusion Criteria

Inclusion criteria are as follows: (1) CMV group was neonates infected with congenital CMV, and healthy or noninfected newborns were as the control group; (2) cohort studies; and (3) English literatures.

Exclusion criteria are as follows: (1) animal experiments; (2) newborns treated after infection; and (3) meta-analyses, reviews, case reports, conference abstracts, and letters.

### 2.3. Quality Assessment and Data Extraction

Two researchers (Zhankui Li, Xiang Han) reviewed the identified literatures and extracted the research data according to inclusion and exclusion criteria. If a discrepancy existed, a third party (Li Zhang) would participate in the extraction of data. For each study, following information was extracted, including author, year, country, gender, gestation weeks or months, and birthweight. Newcastle-Ottawa Scale (NOS) was used to evaluate the literature quality. The scale has a total score of 10, with <5 as medium to low quality and ≥5 as high quality.

The association between congenital CMV infection and brain injury in neonates was assessed by hearing impairment, SNHL, microcephaly, neurodevelopmental delay, mental development index (MDI), and psychomtive development index (PDI).

The Stata 15.0 software (Stata Corporation, College Station, TX, USA) was used for statistical analysis. Heterogeneity test was conducted for each indicator and measured by *I*^2^ statistics. If *I*^2^ ≥ 50%, the random effects model was applied; otherwise, the fixed effects model was used. Sensitivity analysis was performed for all models. Weighed mean difference (WMD) was used as the effect size for measurement data, and risk ratio (RR) was as the effect indicator. *P* < 0.05 was considered statistically significant.

## 3. Results

**Figure 1:**
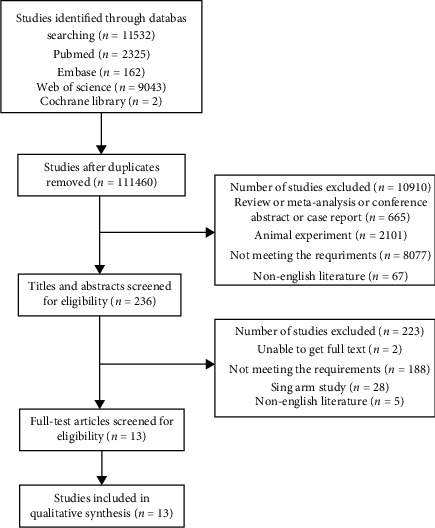
The flow chart of literature search.

Initially, 11,532 potential literatures were searched through database; 11,146 articles were identified after duplicates removed. By checking the titles and abstracts, 236 studies were identified. Finally, 13 full-text articles were screened for eligibility in this meta-analysis, including 4,262 participants with 1,114 neonates in the CMV group and 3,148 neonates in the control group. The flow chart of literature search is shown in Figure 1. The basic characteristics of enrolled studies are summarized in Table 1.

### 3.1. Hearing Impairment

**Figure 2:**
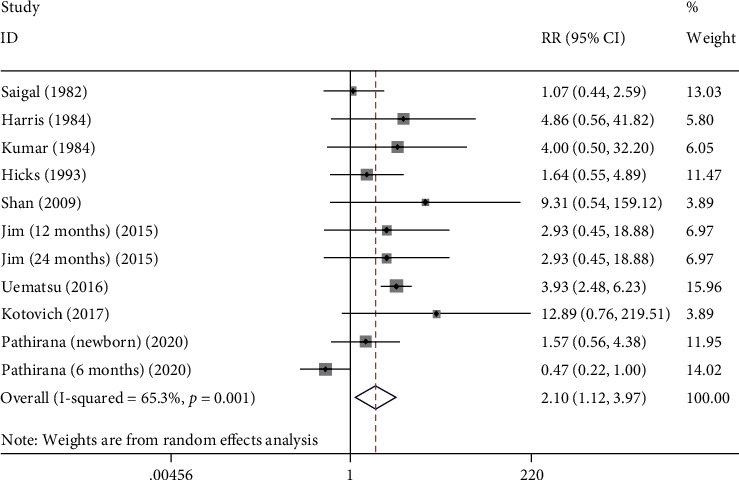
The forest plots of hearing impairment between CMV group and control group.

The hearing impairment was analyzed in 9 studies. The results of heterogeneity test showed that *I*^2^ = 65.3%, so the random effects model was adopted. The results showed that the rate of hearing impairment in CMV group was significantly higher than that in control group (RR: 2.105, 95% CI: (1.115, 3.971), *P* = 0.002) (Figure 2 and Table 2).

**Figure 3:**
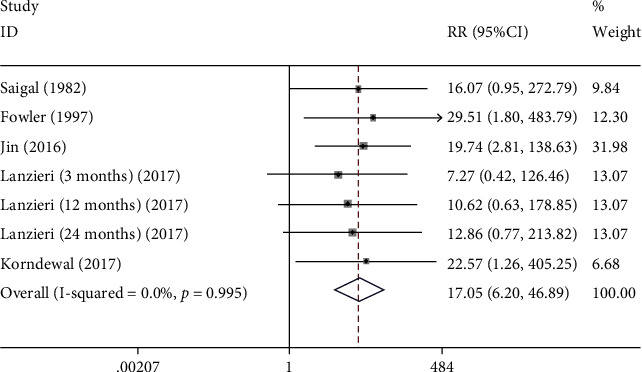
The forest plots of SNHL between CMV group and control group.

A total of 5 studies were enrolled to assess the association between SNHL and congenital CMV infection. Fixed-effect model was adopted (*I*^2^ = 0.0%). It showed that the SNHL rate in the CMV group was higher than that in the control group (RR: 17.051, 95% CI: (6.201, 46.886), *P* < 0.001), indicating SNHL was associated with congenital CMV infection (Figure 3 and Table 2).

**Figure 4:**
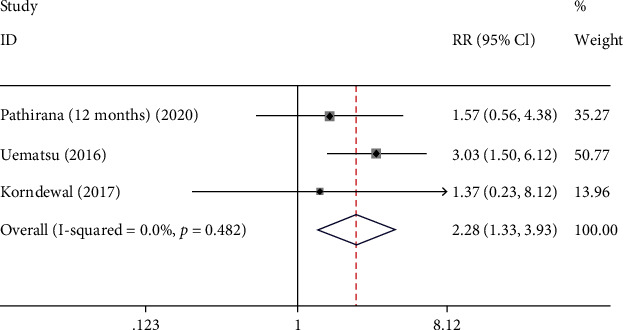
The forest plots of microcephaly between CMV group and control group.

There were 3 studies that were involved to investigate the potential association between microcephaly and congenital CMV infection. The pooled results showed that congenital CMV infection increased the risk of microcephaly in neonates infected with congenital CMV (RR: 2.283, 95% CI: (1.325, 3.935), *P* = 0.003) (Figure 4 and Table 2).

### 3.2. Neurodevelopmental Delay

**Figure 5:**
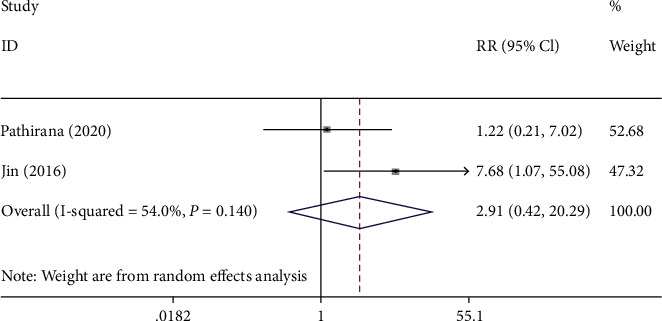
The forest plots of neurodevelopmental delay between CMV group and control group.

The neurodevelopmental delay was identified in 3 studies to assess potential association between congenital CMV infection and neurodevelopmental delay. The results revealed that there was no significant difference in neurodevelopmental delay between the infection group and the control group (*I*^2^ = 54.0%, RR: 2.910, 95% CI: (0.417, 20.285), *P* = 0.281) (Figure 5 and Table 2).

**Figure 6:**
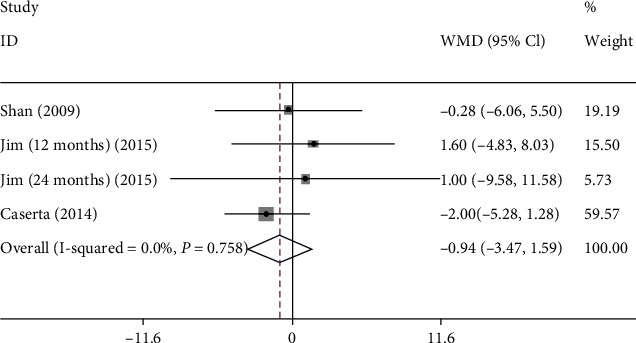
The forest plots of MDI between CMV and control groups.

Totally 3 studies were included in the analysis of association between MDI and congenital CMV infection. The fixed-effect model was used (*I*^2^ = 0.0%). It was shown that MDI in the infection group was no difference compared with the control group (WMD: -0.940, 95% CI: (-3.473, 1.593), *P* = 0.467) (Figure 6 and Table 2).

**Figure 7:**
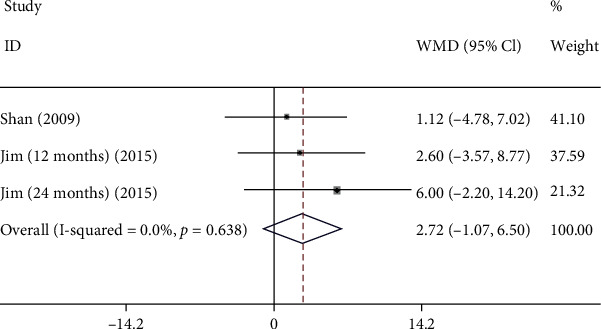
The forest plots of PDI between CMV group and control group.

A total of 2 studies were enrolled to assess association between congenital CMV infection and PDI. The results revealed that congenital CMV infection could not increase the risk of PDI in newborns infected with congenital CMV (WMD: 2.717, 95% CI: (-1.068, 6.501), *P* = 0.159) (Figure 7 and Table 2).

Begg's test was used for the assessment of publication bias, and the results showed that there was no publication bias in hearing impairment (*t* = 0.81, *P* = 0.441). Moreover, the sensitivity analysis for each model was carried out. Sensitivity analysis result proofs that the findings are trustworthy (Table 2).

## 4. Discussion

Brain injury often leads to developmental disorders such as motor function and intelligence in children and even leads to the death of children [13]. Neonatal brain injury causes lifelong morbidity for the survivors, with high emotional costs to the individual and the family plus a heavy economic burden for society. Scarce studies were conducted to investigate brain injury in congenital CMV infection. In this meta-analysis, we explored the association between congenital CMV infection and brain injury in neonates based on a comprehensive search of literatures from a variety of databases. A total of 13 studies involving 3855 participants were enrolled. The results indicated that the risk of hearing impairment, SNHL, and microcephaly in neonates infected congenital CMV were higher than that in the control group. Nevertheless, there was no significant difference in neurodevelopmental delay, MDI, and PDI between CMV group and the control group. The result suggested that congenital CMV infection in neonates is associated with the risk of hearing impairment, SNHL, and microcephaly in neonates.

Our results indicated that congenital CMV infection that increased the risk of hearing impairment in neonates is supported by multiple studies [8, 14, 15]. The exact mechanism of hearing impairment has not been clarified, but several studies have shown that the inflammatory response of the inner ear is more related to the hearing impairment of newborns than the direct damage caused by viruses [16, 17]. Goderis et al. found that the overall incidence of hearing loss in congenital CMV is 12.6%, with the majority of bilateral hearing loss in symptomatic children and unilateral losses predominated in asymptomatic group, indicating that the risk of hearing impairment in neonates is associated with congenital CMV infection [14]. Likewise, Yamamoto et al. [15] have found that congenital CMV is an important cause of permanent hearing impairment in childhood in all settings. A study has have shown that no matter what the cause of hearing impairment is and no matter the impairment is mild or severe, as long as it is found before 6 months after birth and the child's cognitive ability is normal, the child's language ability can basically reach the normal level after systematic and effective intervention [18]. Early detection of hearing impairment in children with CMV infection and systematic and effective clinical treatment are particularly important.

Some studies have suggested that congenital CMV infection is the leading nongenetic cause of SNHL during childhood [8, 19–21]. Our finding showed that SNHL in neonates infected congenital CMV was significantly higher than that in the control group. A meta-analysis by Vries et al. [21], which explore different type of maternal CMV infection during pregnancy, namely, primary (PI) versus nonprimary (NPI), found that the prevalence of SNHL was 13% in the PI group and 11% in the NPI group, respectively. Likewise, previous studies whose population based studies in Sweden [22], Canada [23], and USA [8, 24] have reported that between 9.3% and 17% of infants with congenital CMV infection would have SNHL. The rates of SNHL reported by these studies ranged between 22% and 41% in children with clinically apparent or symptomatic infection and between 6% and 16% in those with subclinical or asymptomatic infection. Yamamoto et al. [25] found that even in populations with near universal immunity to CMV, congenital CMV infection is a significant cause of SNHL. This prompts that comprehensive diagnostic workup for the patient with congenital CMV infection is vital for guiding treatment and intervention options for SNHL.

Microcephaly is generally defined as head circumference ≤ 2 standard deviations below the mean for gestational age [26]. Most microcephaly associated with congenital infections is a reflection of neurotropism for fetal central nervous system (CNS) cells, with massive destruction of neural tissue during the early development of the CNS of the fetus [27–29]. Several studies found that maternal CMV infection is associated with a 30% chance of congenital infection and as much as a 15% chance of clinically apparent manifestations at birth (symptomatic congenital CMV), with up to 50% of these infants manifesting microcephaly [30–32]. Similar results were observed in our study. This suggests that attention should be paid to microcephaly associated with congenital CMV infection in clinical practice as microcephaly is generally accompanied by mental retardation due to severe limitation of brain development.

We did not find association between congenital CMV infection and increasing risk of neurodevelopmental delay. Likewise, a cohort study, aiming to evaluate neurological and growth outcomes in South African children with congenital CMV, found that there was no significant difference in neurodevelopment between cases and controls at 12 months of age [33]. Bartlett et al. did prospective studies of asymptomatic congenital CMV infected children with follow-up of one to 6 years and reported a cumulative incidence of neurodevelopmental impairment between 0% and 9.1% [34]. In contrast, studies of symptomatic newborns reported a prevalence of 30%-50% neurological impairment during childhood [35–37]. Although we did not find the association between congenital CMV infection and increasing risk of neurodevelopmental delay, the contradictory findings suggest us paying attention to the type of fetal infection as it showed symptomatic newborns reported higher risk of neurodevelopmental delay.

Potential confounding factors may have an impact on our result. Nevertheless, most of the articles included studies in this meta-analysis did not adjust confounding factors. Potential confounding factors may be marital status, economic situation, educational background of parents, maternal age at conception, time of maternal CMV infection, whether treated, estimated gestational age at delivery, and neonatal symptoms at birth in this study [2, 9, 38–40]. Age is a common confounding factor. Using survival analysis, a study [9] found that the proportion of SNHL in children with congenital CMV infection increased from 7% at age 3 months to 14% at age 5 years and 25% at age 18 years among case-patients and from 0% at age 5 years to 8% at age 18 years among controls. More studies will be important to confirm these findings and inform future guidance on the optimal duration of audiologic monitoring for children with congenital CMV infection. Persons of lower socioeconomic status tended to report higher risk of CMV than those in the general population [38]. This contrasts with the conclusions of Preece et al. [39], who reported once age, race, and marital status had been taken into account there was no difference in the estimated prevalence of congenital CMV between infants born to mothers from manual and nonmanual social class. Besides, the expected number of infected newborns among those in worse clinical conditions is higher than in the general population, once the deleterious effects determined by the virus begin in the fetus [40]. Notably, the birth prevalence was lower in infants born to mothers who were ascertained in the prenatal period than in infants born to mothers who were ascertained at delivery. The lower birth prevalence among women ascertained prenatally may occur because women seeking prenatal care tend to have lower risk for a variety of poor outcomes [2]. These potential confounding variables suggest that interventions might be most efficient if they are targeted toward certain groups. More original studies are needed in the future to further elucidate the association between congenital CMV infection and neonatal brain injury.

The strengths of the current study need to be mentioned. This was the first attempt based on comprehensive databases to investigate association between congenital CMV infection and brain injury in neonates. Besides, all the studies were cohort studies, making the findings more trustworthy. However, some limitations of our study must be acknowledged. First, in our study, we estimated the risk of brain injury associated with congenital CMV infection, but we did not estimate the specific maternal infection or fetal infection subtypes. In future study, we will do further study based on maternal infection or fetal infection to investigate association between congenital CMV infection and brain injury in newborn. Second, most of included studies were retrospective studies, which may lead to recall bias. Third, residual confounding variables are a problem. Uncontrolled or unmeasured confounding factors have the potential for bias; the possibility that residual confounders influenced the results cannot be ruled out. Furthermore, for risk estimates of brain injury associated with congenital CMV infection, the results mainly relied on the 13 total studies, so more studies should be included in future studies to provide further support for our results.

## 5. Conclusion

This meta-analysis explored association between congenital CMV infection and brain injury in neonates based on comprehensive databases. The result suggested the risks of hearing impairment, SNHL, and microcephaly in neonates during childhood may be associated with congenital CMV infection. In clinical application, do early screening of congenital CMV infection to improve the detection rate of infected children, reducing the incidence of sequelae and improving the prognosis, finally bringing significant social benefits to improve the quality of the population.

## Figures and Tables

**Table 1:** Baseline characteristics of included study.

Author	Year	Country	Case				Control			
			Total	M/F	Gestation, weeks/months	Birthweight/g	Total	M/F	Gestation, weeks/months	Birthweight, g
Saigal	1982	Canada	47	30/17	39 ± 2.2 w	3130 ± 657	46	29/17	39 ± 2.5 w	3120 ± 645 g
Harris	1984	Sweden	42				51			
Kumar	1984	USA	17		7.6 (6.5-10.9) m		21		7.4 (6.5-10.9) m	
Hicks	1993	USA	34				2002			
Fowler	1997	USA	307	158/149			201	90/111		
Shan	2009	China	52				21			
Jim	2015	China	14		27.9 ± 2.6 w	1093.7 ± 251.4	41		29.2 ± 2.5	1153.7 ± 235.7 g
Uematsu	2016	Japan	54	23/31	39 (36-41) w	2634 (2497-2771)	106	53/53	38 w (23-42 w)	2516-2783 g
Jin	2016	USA	186	96/90			51	35/16		1750-4170 g
Korndewal	2017	The Netherlands								
Kotovich	2017	Israel	90		33.1 ± 2.0 w		199		31.8 ± 2.3 w	
Lanzieri	2017	USA	92	53/39			51	37/14		
Pathirana	2020	South Africa	46	25/21	38 (36-40) w	2845 (2455-3190)	84	43/41	38 (36-39) w	2902.5 (2595-3195) g

**Table 2:** Overall results and sensitivity analysis.

Outcomes	RR, 95% CI	*P*	*I* ^2^
Hearing impairment			
Overall	2.105 (1.115, 3.971)	0.022	65.3%
Sensitivity analysis	2.105 (1.115, 3.971)		
SNHL			
Overall	17.051 (6.201, 46.886)	<0.001	0.0%
Sensitivity analysis	17.051 (6.201, 46.886)		
Microcephaly			
Overall	2.283 (1.325, 3.935)	0.003	0.0%
Sensitivity analysis	2.283 (1.325, 3.935)		
Neurodevelopmental delay			
Overall	2.910 (0.417, 20.285)	0.281	54.0%
Sensitivity analysis	2.910 (0.417, 20.285)		
MDI			
Overall	-0.940 (-3.473, 1.593)	0.467	0.0%
Sensitivity analysis	-0.940 (-3.473, 1.593)		
PDI			
Overall	2.717 (-1.068, 6.501)	0.159	0.0%
Sensitivity analysis	2.717 (-1.068, 6.501)		

SNHL: sensorineural hearing loss; MDI: mental development index; PDI: psychomtive development index.

## Data Availability

The data utilized to support the findings are available from the corresponding authors upon request.
